# Complete Genome Sequence of *Mycoplasma canadense* Strain HAZ 360_1 from Bovine Mastitic Milk in Japan

**DOI:** 10.1128/genomeA.00984-14

**Published:** 2014-10-02

**Authors:** Eiji Hata

**Affiliations:** Dairy Hygiene Research Division, National Institute of Animal Health (NIAH), National Agriculture and Food Research Organization (NARO), Sapporo, Hokkaido, Japan

## Abstract

Bovine mycoplasmal mastitis is spreading quickly among cows. *Mycoplasma canadense*, a causal species of bovine mastitis, reduces milk quality and quantity via the infiltration of numerous inflammatory cells. Presented here is the complete 693,241-bp genome sequence of *M. canadense* strain HAZ 360_1, which was isolated in Japan.

## GENOME ANNOUNCEMENT

*Mycoplasma canadense* is a causal bacterium of bovine mastitis, and it is also isolated from the respiratory and reproductive tracts of cattle (1, 2). An intramammary *M. canadense* infection results in an immediate and sharp decrease in milk yield, a massive amount of inflammatory cells infiltrating the milk of all quarters, and persistent infection for long periods (2). The clinical picture is that of a mild outbreak of *Mycobacterium bovis* mastitis (1–3). Despite the importance of *M. canadense*, little genetic information on *M. canadense* is available. The entire genome sequence of *M. canadense* strain HAZ 360_1, isolated in 2008 from bovine mastitic milk in Japan, is presented here.

Total genomic DNA was prepared from *M. canadense* strain HAZ 360_1 and subjected to 454 Titanium sequencing at Hokkaido System Science Co. (Sapporo, Japan). The resulting reads were assembled *de novo* using the GS *de novo* Assembler software version 2.7 (Roche), yielding 41 contigs with 96.7× coverage. An analysis of the contig ends, together with polymerase chain reaction (PCR) amplification and amplicon cloning, showed that the 693,241-bp genome has a closed-ring structure. After the initial automated annotation performed using Microbial Genome Annotation Pipeline version 2.18 at the DNA Data Bank of Japan (http://migap.ddbj.nig.ac.jp/mgap/jsp/index.jsp) (4–6), manual curation was performed, followed by the verification of potential pseudogenes by PCR and Sanger sequencing. As a result, 484 open reading frames, 15 pseudogenes, 32 tRNAs, and two sets of each rRNA (5S rRNA, 16S rRNA, and 23S rRNA) were confirmed in this genome sequence. The G+C content is 24.34%.

As anticipated based on its 16S rRNA-based phylogeny, most genes in *M. canadense* strain HAZ 360_1 exhibit high similarity to the amino acid sequences of the genes carried by members of the *Mycoplasma alkalescens* cluster, with the best similarity shown with genes from *Mycoplasma arginini* (7).

The enzymes involved in the arginine hydrolysis pathway and acetate kinase have an important role in the generation of energy in arginine-metabolizing mycoplasmas, such as *M. alkalescens*, and genes coding for these enzymes were found in the genome sequence of *M. canadense* (8, 9). However, the genes of proteins involved in the synthesis of capsular polysaccharides and the production of active oxygen-containing molecules, which are suggested to be important mycoplasmal etiologic agents (e.g., UTP-glucose-1-phosphate-uridyltransferase and glycerol-3-phosphate oxidase) were not confirmed (10, 11).

A part of the amino acid sequences of the hypothetical proteins MCAN 360_0280, MCAN 360_0281, and MCAN 360_0504 showed certain similarities to the surface proteins involved in the antigenic variation shift in other mycoplasma species (11, 12). Moreover, the discriminative homopolymeric tract of contiguous adenines [poly(A)] is located upstream of the repetitive regions in these hypothetical proteins (11, 12). These genes contain homologous regions, which consist of 81 periodic amino acid sequences.

The genomic sequence of *M. canadense* will provide a foundation for further investigations of this species, and it is hoped that this study will contribute to the reduction of bovine diseases, such as mastitis.

### Nucleotide sequence accession number.

The whole-genome sequence has been registered at DDBJ/EMBL/GenBank under the accession no. AP014631.
